# A Ketogenic Diet in Combination with Gemcitabine Mitigates Pancreatic Cancer-Associated Cachexia in Male and Female KPC Mice

**DOI:** 10.3390/ijms241310753

**Published:** 2023-06-28

**Authors:** Natalia E. Cortez, Suraj Pathak, Cecilia Rodriguez Lanzi, Brian V. Hong, Ryman Crone, Rasheed Sule, Fangyi Wang, Shuai Chen, Aldrin V. Gomes, Keith Baar, Gerardo G. Mackenzie

**Affiliations:** 1 Department of Nutrition, University of California, One Shields Ave., Davis, CA 95616, USA; natcortezp@ucdavis.edu (N.E.C.); mcrod@ucdavis.edu (C.R.L.); bvhong@ucdavis.edu (B.V.H.); 2 Department of Physiology and Membrane Biology, One Shields Ave., Davis, CA 95616, USA; sjpathak@ucdavis.edu (S.P.); rrcrone@ucdavis.edu (R.C.); rosule@ucdavis.edu (R.S.); avgomes@ucdavis.edu (A.V.G.); kbaar@ucdavis.edu (K.B.); 3 Department of Neurobiology, Physiology and Behavior, University of California, One Shields Ave., Davis, CA 95616, USA; 4 Department of Animal Science, University of California, One Shields Ave., Davis, CA 95616, USA; faywang@ucdavis.edu; 5 Division of Biostatistics, Department of Public Health Sciences, University of California, One Shields Ave., Davis, CA 95616, USA; shschen@ucdavis.edu; 6 University of California Davis Comprehensive Cancer Center, Sacramento, CA 95817, USA

**Keywords:** cachexia, pancreatic cancer, ketogenic diet, gemcitabine, cancer-associated cachexia

## Abstract

Cancer-associated cachexia (CAC) is a critical contributor to pancreatic ductal adenocarcinoma (PDAC) mortality. Thus, there is an urgent need for new strategies to mitigate PDAC-associated cachexia; and the exploration of dietary interventions is a critical component. We previously observed that a ketogenic diet (KD) combined with gemcitabine enhances overall survival in the autochthonous LSL-KrasG12D/+; LSL-Trp53 R172H/+; Pdx1-Cre (KPC) mouse model. In this study, we investigated the effect and cellular mechanisms of a KD in combination with gemcitabine on the maintenance of skeletal muscle mass in KPC mice. For this purpose, male and female pancreatic tumor-bearing KPC mice were allocated to a control diet (CD), a KD, a CD + gemcitabine (CG), or a KD + gemcitabine (KG) group. We observed that a KD or a KG-mitigated muscle strength declined over time and presented higher gastrocnemius weights compared CD-fed mice. Mechanistically, we observed sex-dependent effects of KG treatment, including the inhibition of autophagy, and increased phosphorylation levels of eIF2α in KG-treated KPC mice when compared to CG-treated mice. Our data suggest that a KG results in preservation of skeletal muscle mass. Additional research is warranted to explore whether this diet-treatment combination can be clinically effective in combating CAC in PDAC patients.

## 1. Introduction

Cancer-associated cachexia (CAC) is a complex metabolic disorder characterized by the unintentional and ongoing wasting of skeletal muscle (SKM), with or without loss of adipose tissue [1,2]. Patients with pancreatic ductal adenocarcinoma (PDAC) are among those with the highest incidence of CAC, estimated at approximately 80% [3,4], and this contributes directly to the mortality from this disease [5]. Paradoxically, the bidirectional interaction of cachexia with chemotherapy further complicates the scenario. While CAC is a critical contributor to chemotherapy mortality, chemotherapeutic agents can, in turn, aggravate cachexia [6,7]. Hence, the loss of SKM mass while receiving PDAC-treatment is considered a predictor of poor prognosis [8]. Unfortunately, to date, there are no approved therapeutics recognized to effectively reverse or manage CAC [2]. Therefore, therapies that support the preservation of muscle mass during PDAC-associated cachexia are warranted. Evidence on the impact of nutritional support on cachexia is limited [9], but beneficial effects of enteral nutrition support on weight and lean body mass stability in pancreatic cancer patients with cachexia was demonstrated [10], and novel nutritional interventions are worth exploring. 

Ketogenic diets (KDs), which are high fat/low carbohydrate dietary regimens aimed to produce ketosis, were shown to exert a protective effect against muscle mass loss [11]. In aging mice, KDs promote the preservation of muscle function and slow sarcopenia [12]. The metabolic alterations induced by KDs, particularly the production of ketone bodies, are associated with lower SKM protein degradation, decreased secretion of pro-inflammatory cytokines, and fewer metabolites involved in the pathogenesis of CAC [13,14]. However, the impact of a KD in PDAC-associated cachexia is limited, with only one study showing that muscle wasting in a PDAC orthotopic model was improved with a KD [15]. 

We recently documented that a ketogenic diet (KD) combined with gemcitabine enhances overall survival in the autochthonous *LSL-KrasG12D/+*; *LSL-Trp53 R172H/+*; *Pdx1-Cre* (KPC) mouse model [16]. It is noteworthy that KPC mice develop pancreatic tumors that recapitulate many of the histopathological, genomic, and clinical features of human PDAC, including the development of progressive cachexia, which makes them an appropriate model to evaluate strategies to mitigate this complication of PDAC [17]. We hypothesized that the increase in overall survival might be, in part, due to a mitigation of CAC by KD. To test this hypothesis, we performed a secondary analysis with the overall goal of evaluating the effect of feeding a strict KD alone or in combination with gemcitabine on the maintenance of skeletal muscle mass in KPC mice and the potential cellular mechanisms underlying the benefits. Although sex-specific differences in PDAC-associated cachexia may exist, the limited research in this area further restricts the development of effective treatments [18]. Therefore, we also evaluated the impact of biological sex on the response to a KD combined with chemotherapy during PDAC-associated cachexia. We observed that a KD in combination with gemcitabine preserves muscle strength in KPC mice, in part, by decreasing autophagy and increasing phosphorylation levels of eIF2α, with female KPC mice demonstrating a greater effect than males. 

## 2. Results

### 2.1. A Ketogenic Diet, Alone or in Combination with Gemcitabine, Preserves Muscle Strength in KPC Mice

**Figure 1:**
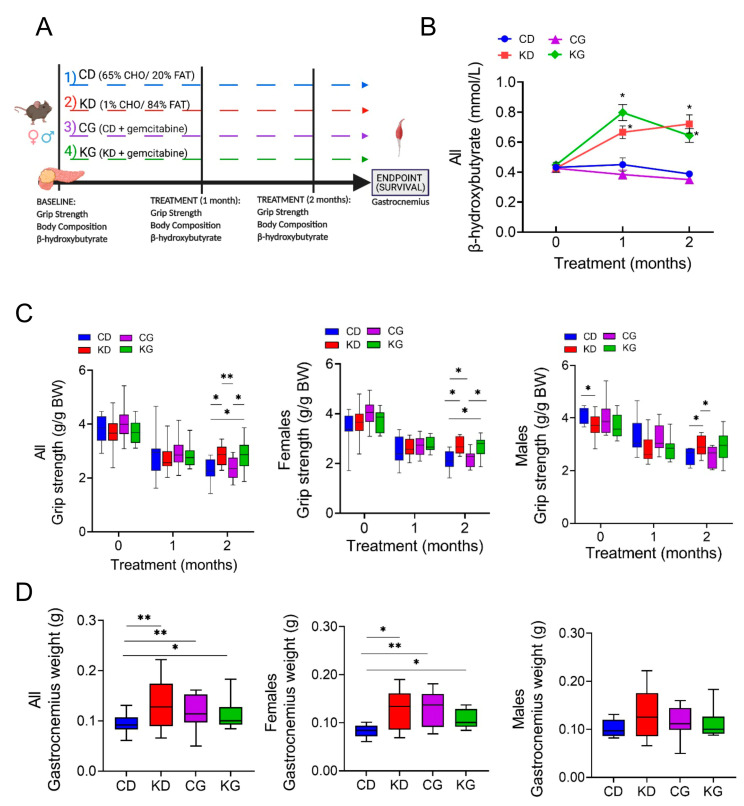
A ketogenic diet alone and in combination with gemcitabine preserves muscle strength and weight in KPC mice. (**A**) Schematic outline of the survival study design. Once pancreatic tumors were detected, female and male KPC mice were randomized to a control diet (CD), ketogenic diet (KD), CD plus gemcitabine (CG) or KD plus gemcitabine (KG) (*n* = 16–23/group) until endpoint. (**B**) Non-fasted blood β-hydroxybutyrate levels at baseline, one and two months after diet initiation. (**C**) Relative forelimb-grip strength force at baseline, one and two months after diet initiation and (**D**) gastrocnemius weight are shown for all (left), females only (center) and males only (right) KPC mice fed a control diet (CD), ketogenic diet (KD), CD plus gemcitabine (CG) or KD plus gemcitabine (KG). Values are expressed as mean ± SEM; * *p* < 0.05, ** *p* < 0.01.

Once tumors were detected, male and female KPC mice were randomized into control diet (CD), ketogenic diet (KD), CD + gemcitabine (CG), or KD + gemcitabine (KG) groups (16–23 mice/group) until they reached their endpoint (Figure 1A). As expected, after one month on the diet, KD- and KG-fed mice had higher post-prandial blood β-hydroxybutyrate (β-HB) levels compared to mice fed a CD or a CG (Figure 1B). This increase was observed in both female and male mice that were fed a KD or KG (Figure S1). When evaluating overall survival, only KG significantly increased overall median survival (119 days) by 42%, compared to CD-fed KPC mice (80 days). Of note, KPC mice fed a KD alone (94 days) or CD treated with GEM (88 days) were unable to significantly extend overall survival compared to CD-fed mice [16].

Given that KD in combination with gemcitabine enhances overall survival in the KPC mice [16], we hypothesized that the increase in overall survival might be, in part, due to a mitigation of CAC by KD. For this purpose, we investigated the effect and mechanisms of feeding a strict KD alone or in combination with gemcitabine on the maintenance of skeletal muscle mass in KPC mice. First, we assessed the impact of a KD ± gemcitabine on muscle strength in male and female KPC mice, using the forelimb grip-strength test. KPC mice fed a CD, either alone or in combination with gemcitabine, showed weakening after two months of treatment, compared to baseline levels (Figure 1C). In contrast, KPC mice fed a KD, either alone or combined with gemcitabine, better maintained muscle strength after 2 months, compared with mice fed a CD. When analyzed by sex, muscle strength was significantly higher in the CD males than in KD at baseline, yet after two months on the diet, the opposite was observed, with KD-fed mice having greater muscle strength (Figure 1C). In females, both KD and KG had significantly greater muscle strength than CD-fed groups. 

The gastrocnemius (GTN) muscle mass at euthanasia was higher in KPC mice treated with KD or KG. Interestingly, even though gemcitabine had no effect on strength, the CG group did show greater GTN mass when compared to CD mice (Figure 1D). When separated by sex, a positive effect of the intervention was only observed in females (Figure 1D). 

We then conducted linear regression models for grip strength adjusted by sex and age at the start of treatment and observed a significant effect of sex on grip strength performance (*p* < 0.001). To investigate whether a KD ± gemcitabine’s effect increases over time, we performed linear mixed-effects regressions. Both ketone bodies and grip strength determinations were fitted for repeatedly measured outcomes (measured at baseline, and 1 and 2 months after the start of the intervention), which included fixed effects for diet, drug, time, and their 2-way and 3-way interactions. As shown in Table 1, a significant diet-time interaction for grip strength was detected, indicating that the effect of a KD and a KD in combination with gemcitabine increases over time (1-Month KD vs. CD effect est. = −0.17 g/g BW, *p* = 0.12; 2-Month KD vs. CD effect est. = 0.45 g/g BW, *p* < 0.001). However, the estimated effect of gemcitabine was the same over time and across diet groups (est. = 0.07 g/g BW, *p* = 0.44). 

### 2.2. Effect of a Ketogenic Diet Alone and in Combination with Gemcitabine on Food Intake, Body Weight and Composition in KPC Mice

**Figure 2:**
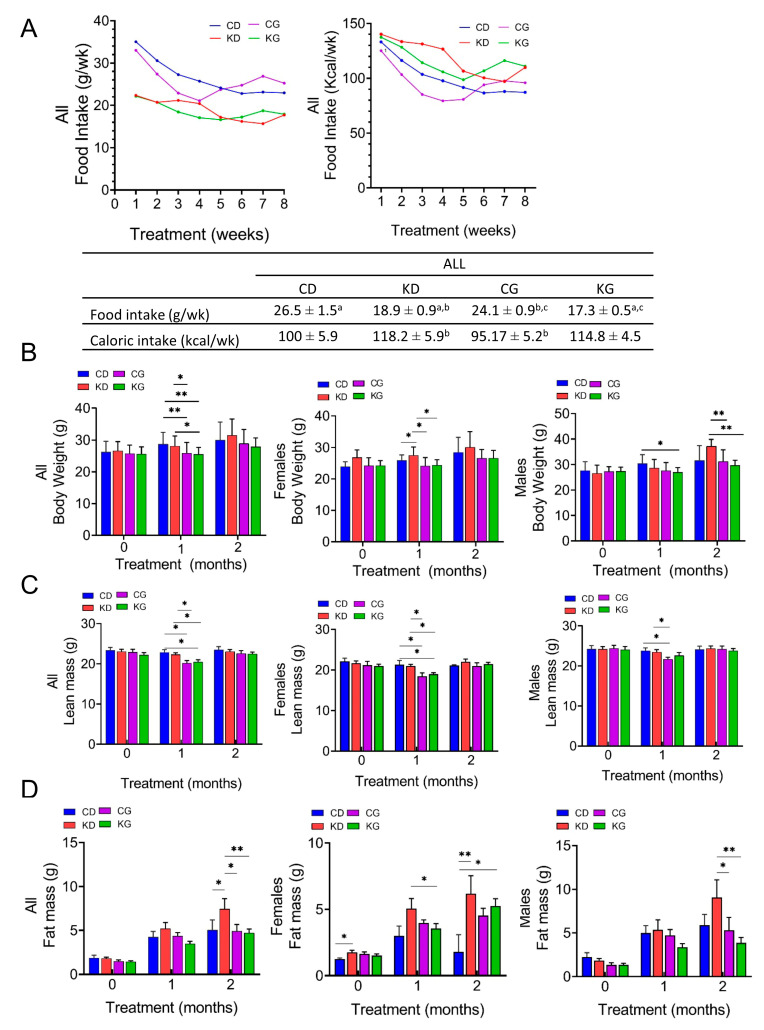
Effect of a ketogenic diet alone and in combination with gemcitabine on food intake, body weight and composition in KPC mice. (**A**) Food intake in grams per week (g/wk) and kilocalories per week (Kcal/wk) during 8 weeks of treatment. Values having different superscripts are significantly different (*p* < 0.05). (**B**) Body weight progression, (**C**) lean mass and (**D**) fat mass of KPC mice fed a control diet (CD), ketogenic diet (KD), CD plus gemcitabine (CG) or KD plus gemcitabine (KG) are shown at baseline, one and two months after diet initiation for all mice (left), females only (center) and males only (right). Values are expressed as mean ± SEM; * *p* < 0.05, ** *p* < 0.01.

Although weekly mean intake in grams (g/wk) was significantly higher for CD (26.5 ± 1.5) and CG (24.1 ± 0.9) than for KD (18.9 ± 0.9) and KG (17.3 ± 0.5), the weekly caloric intake (Kcal/wk) was higher for KD (118.2 ± 5.9) and KG (114.8 ± 4.5) than for CD (100.5 ± 5.9) and CG (95.2 ± 5.2) (Figure 2A). Moreover, the caloric intake was significantly different for the CG group when compared to KD or KG (*p* < 0.05). When the data were disaggregated by sex, the feeding differences were observed in females but not in males (Figure S2). 

We also measured the total body weight (BW) and the body composition of mice at baseline and monthly on intervention. One month after starting the interventions, treatment with gemcitabine led to a reduction in BW (Figure 2B). Mice in the CG and KG groups had lower weights one-month post-treatment compared to those in the CD and KD groups, but the weight was recovered by two months, with no BW differences among the four groups. When data were disaggregated by sex, after 1 month of treatment, a significantly higher BW was observed in female KD mice compared to CD and both gemcitabine-treated groups, with no BW differences among the four groups observed at two months. In males, KG was lower than CD one-month after treatment, but not after two months. The BW of KD-fed mice was significantly higher than CG and KG at two months only in males (Figure 2B).

Regarding body composition, a significant decrease in lean mass was observed in the CG and KG groups compared to both non-gemcitabine groups only at one month (Figure 2C). When analyzed by sex, such effect persisted in females, but in males, a significant decrease was observed only in the CG group and not in the KG group when compared to non-gemcitabine groups. After two months of treatment, significant increases in fat mass were observed in the KD group when compared to all other groups (Figure 2D). In females, the fat mass of KD and KG groups were higher than CD, whereas in males, fat mass of the KD group was significantly higher than both gemcitabine groups at the two-month time point.

### 2.3. A Ketogenic Diet in Combination with Gemcitabine Increases Blood Ketone Bodies in KPC Mice

**Figure 3:**
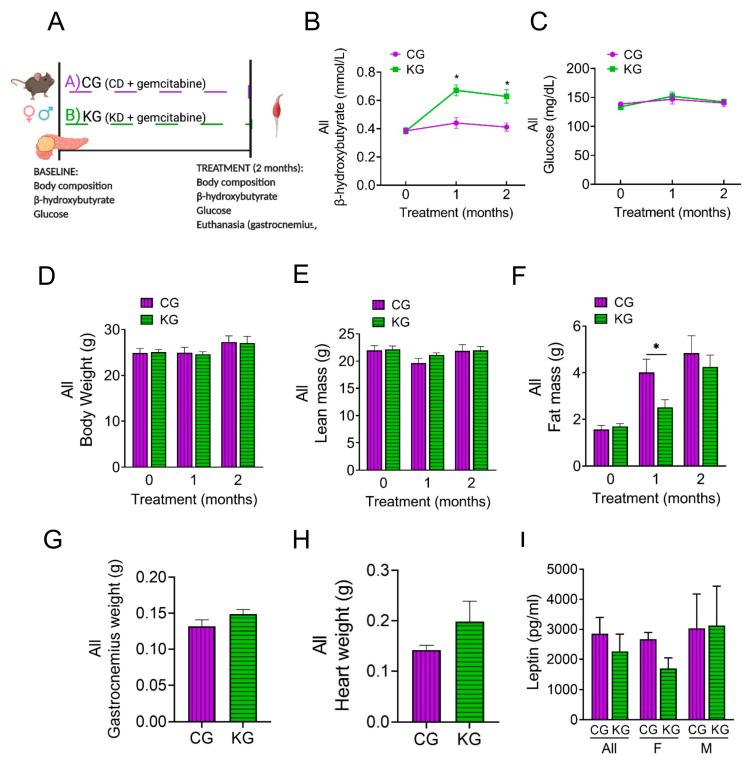
A ketogenic diet in combination with gemcitabine induces metabolic changes in KPC mice. (**A**) Schematic outline of the mechanistic study design. Female and male KPC mice were randomized to a control diet plus gemcitabine (CG) or to a ketogenic diet plus gemcitabine (KG) and euthanized after 2 months; *n* = 4 per sex/group. (**B**) Non-fasted blood β-hydroxybutyrate levels, (**C**) circulating levels of non-fasting glucose, (**D**) body weight, (**E**) lean mass, and (**F**) fat mass progression are shown at baseline, one and two months after diet plus gemcitabine initiation of KPC mice treated with CG or KG. (**G**) Gastrocnemius weight, (**H**) heart weight, and (**I**) serum leptin levels at euthanasia shown for CG- and KG-treated KPC mice. Values are expressed as mean ± SEM; * *p* < 0.05.

To elucidate potential mechanisms underlying the improved muscle mass and strength on a KD when combined with gemcitabine, we conducted a mechanistic study in which three-month-old male and female KPC mice bearing pancreatic tumors were treated with gemcitabine while being fed either a control (CG) or a ketogenic (KG) diet for 2 months (Figure 3A). 

KG-treated mice had increased blood β-HB levels compared to CG mice (Figure 3B). In contrast, no differences in glucose levels were observed between CG and KG groups (Figure 3C). Although ketone bodies were increased, no differences in the levels of ketone body metabolic enzymes succinyl CoA: 3-oxoacid CoA transferase (OXCT1), 3-hydroxybutyrate dehydrogenase 1 (BDH1), and acetyl-CoA acetyltransferase 1 (ACAT1) in GTN muscles were observed between CG and KG groups (Figure S3). 

After 2 months of treatment, no differences were observed in either BW or lean mass as a function of diet (Figure 3D,E). With fewer groups to correct for, the CG group demonstrated a significantly greater fat mass at one month; however, by two months, the groups had similar fat mass (Figure 3F). The GTN weight was similar between the CG and KG groups at the 2-month time point (Figure 3G). Moreover, heart weight was obtained as a marker of cachexia and was not significantly different between CG and KG-treated mice (Figure 3H). 

We next assessed serum leptin levels. Although no significance differences were observed between KG and CG groups, a trend for lower leptin levels (pg/mL) was observed for KG females (1693 ± 354) when compared to KG males (3216 ± 1312) (*p* = 0.24) (Figure 3I).

### 2.4. A Ketogenic Diet Does Not Affect Inflammatory Cytokine Levels in KPC Mice Treated with Gemcitabine

**Figure 4:**
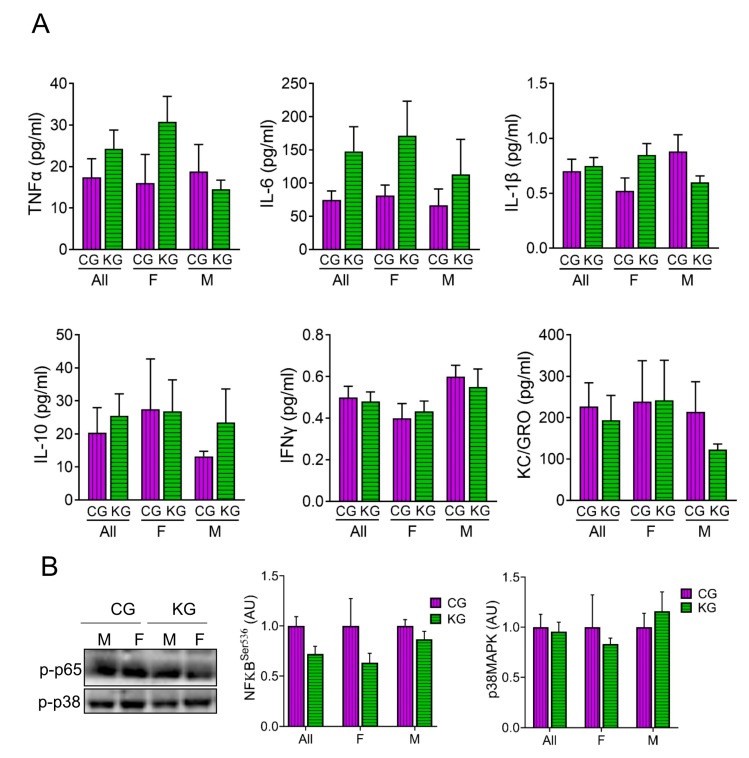
Effect of a ketogenic diet in combination with gemcitabine on serum cytokine levels and inflammatory markers in KPC mice. (**A**) Levels of pro-inflammatory cytokines TNFα, IL-6, IL-1β, IL-10, IFN-γ, and KC/GRO were determined in serum obtained from CG- and KG-treated female and male KPC mice following 2 months of treatment; *n* = 4 per sex/group; values are expressed as mean ± SEM. (**B**) Immunoblots of p-p65 and p-p38 proteins in gastrocnemius homogenates isolated from CG- and KG-treated female and male KPC mice following 2 months of treatment. *n* = 2–5 per sex/group; values are expressed as mean ± SEM.

We assessed the levels of several cytokines involved in inflammation in serum of male and female KPC mice treated with KG or CG for two months. As shown in Figure 4, diet did not result in significant changes to circulating IL-6, TNF-α, IL-1β, IFN-γ, IL-10, KC/GRO, nor MCP-1 levels. Moreover, stratifying by sex had no effect on cytokine levels (Figure 4A). Even without changes in cytokine levels across diet, the phosphorylation of the inflammatory signaling molecule NFκB was lower in the KG mice (Figure 4B). When stratified by sex, the greatest effect was seen in the females. The phosphorylation of the inflammatory signaling protein p38MAPK was not different between the CG and KD groups.

### 2.5. A Ketogenic Diet Does Not Change Anabolic Signaling in the Gastrocnemius of KPC Mice Treated with Gemcitabine

**Figure 5:**
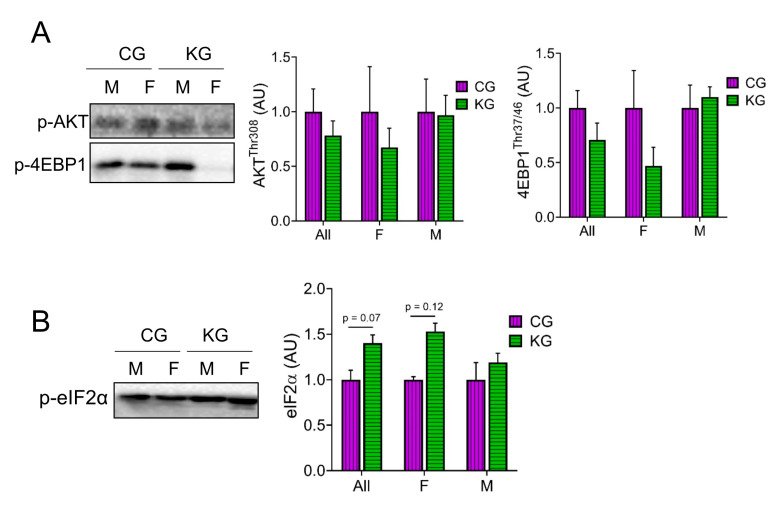
Effect of a ketogenic diet in combination with gemcitabine on skeletal muscle AKT and mTOR signaling. Immunoblots of (**A**) p-4EBP1, p-AKT, and (**B**) p-eIF2α proteins in gastrocnemius homogenates isolated from CG- and KG-treated female and male KPC mice following 2 months of treatment. *n* = 2–5 per sex/group; values are expressed as mean ± SEM.

Next, we analyzed proteins within the Akt/mTOR signaling pathway [14,19,20,21,22,23]. Even though there were no statistically significant differences in the phosphorylation of any of the proteins assayed, there was a consistent pattern where all phosphorylation sites were lowered in the female mice on a KD (Figure 5A). One of the most consistent findings in muscle exposed to a KD was an increase in the phosphorylation of eIF2α [24,25,26]. In GTN muscles of KPC mice treated with gemcitabine, a KD significantly increased (main effect of diet) the phosphorylation of eIF2α and this effect was greatest in females (Figure 5B).

### 2.6. A Ketogenic Diet Alters Proteosome Activity and Autophagy in the Gastrocnemius of KPC Mice Treated with Gemcitabine

**Figure 6:**
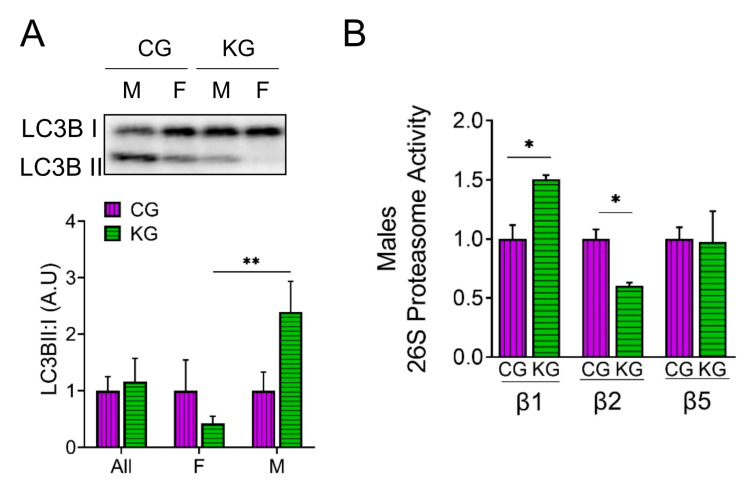
A ketogenic diet in combination with gemcitabine induces autophagy and proteasome activity in the gastrocnemius of KPC mice. Immunoblots of (**A**) LC3B-II, LC3B-I, and (**B**) ATP-dependent (26S) proteasome activity assay of subunit β1, β2, and β5 in gastrocnemius homogenates isolated from CG- and KG-treated female and male KPC mice following 2 months of treatment. Values are expressed as means ± SEM; *n* = 2–5 per sex/group. Values are expressed as mean ± SEM; * *p* < 0.05; ** *p* < 0.01.

We next determined the effect of diet on the amount of LC3B-II and I to estimate autophagy and directly measured proteosome activity in the GTN of CG- and KG-treated KPC mice. An increase in the LC3B-II to LC3B-I ratio was observed in male KG-treated mice when compared to CG-males, whereas the ratio decreased in KG females, suggesting a sex-specific activation of autophagy in males and inhibition of autophagy in females on a KD (Figure 6A). We next evaluated the effect of KG on protein degradation through the ubiquitin–proteasome system (UPS), by measuring the activities of the 3 catalytic subunits of the constitutive proteasome: β1 (caspase-like), β2 (trypsin-like), and β5 (chymotrypsin-like) in male mice. In male mice, treatment with KG led to an increase in the activity of the β1 subunit and a decrease in β2 subunit activity when compared to CG group, whereas no differences in β5 activity were observed between the groups (Figure 6B). Consistent with the relatively minor changes in protein degradation observed, levels of the muscle-atrophy-related proteins MaFBx/atrogin-1 and muscle RING finger 1 (MuRF1) did not differ neither as a function of diet nor when stratified by sex (Figure S4).

### 2.7. Effect of a Ketogenic Diet on Protein Acetylation, Antioxidant Levels and Mitochondrial Proteins in the Gastrocnemius of KPC Mice Treated with Gemcitabine

**Figure 7:**
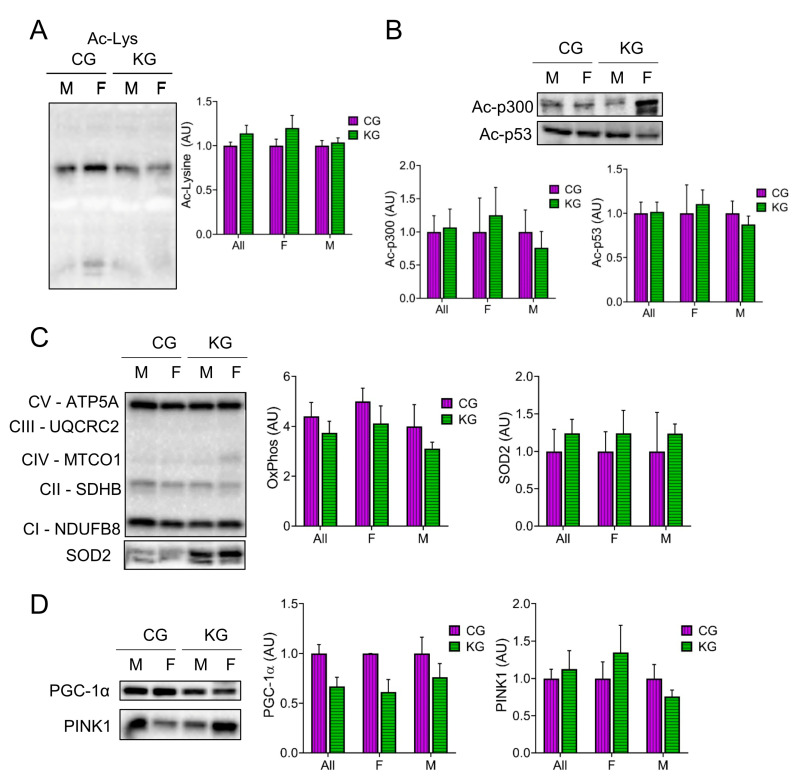
Effect of a ketogenic diet in combination with gemcitabine on skeletal muscle protein acetylation and oxidative metabolism. Immunoblots of (**A**) acetyl-lysine, (**B**) Acetyl- acetyltransferase p300 and Ac-p53, (**C**) oxidative phosphorylation (OXPHOS) proteins from each complex (C-I to C-V), and SOD2, and (**D**) PGC-1α and PINK1 proteins in gastrocnemius homogenates isolated from CG- and KG-treated female and male KPC mice following 2 months of treatment. *n* = 2–5 per sex/group; values are expressed as mean ± SEM.

Since β-HB can inhibit histone deacetylases (HDACs) [27,28] and this can improve muscle function [24], we determined total acetyl-lysine levels in GTN muscles after two months on diet. Total acetyl-lysine levels were not different between the CG and KG groups (Figure 7A). A similar pattern was seen in the acetylation of the acetyltransferase p300 and the tumor suppressor protein p53, where the female mice tended to show higher levels than the males (Figure 7B). Since acetylation is important in the regulation of muscle metabolism, we next determined the effect of diet on mitochondrial mass and signaling. Surprisingly, there was no increase in the level of representative proteins within the electron transfer chain (oxidative phosphorylation; OXPHOS) after 2 months on a KD (Figure 7C), nor in the antioxidant enzyme superoxide dismutase levels (Figure 7C). Furthermore, the mitochondrial regulator PGC-1α tended to decrease in the KD groups (Figure 7D). The maintenance of mitochondria with a decrease in PGC-1α may reflect changes in mitochondrial turnover rates. To estimate the rate of mitophagy, we measured Pink1 levels in the GTN muscles. Pink1 levels were not different between the CG and KG groups (Figure 7D). 

## 3. Discussion

Cancer-associated cachexia is a major complication for PDAC patients, as it impairs treatment tolerance, diminishes quality of life, and reduces overall survival [29]. Thus, there is an active search for interventions that may reduce PDAC-associated cachexia. In this study, we evaluated whether feeding a KD, with or without gemcitabine, mitigated CAC in tumor-bearing KPC mice, a model that replicates the progressive detriment of muscle strength characteristic of PDAC-associated cachexia in humans [30]. 

Previous studies support the notion that KDs could be beneficial for preserving muscle strength [12]. For example, in aged mice, Wallace et al. observed that a KD resulted in preservation of SKM and increased markers of mitochondrial biogenesis, oxidative metabolism, and oxidative stress response, while decreasing protein synthesis and proteasome activity [26]. In an orthotopic xenograft model of pancreatic cancer, a KD fed for three weeks to female nude mice led to significantly diminished cachexia [15]. Although such findings were promising, the effects of a long-term KD in clinically relevant models of PDAC-associated cachexia in combination with chemotherapy were not reported. We observed that a KD concomitant with gemcitabine slowed the rate of muscle strength decline. Consistent with our findings, a KD previously demonstrated increased muscle function and weight preservation in old mice [26]. In a model of colon cancer, muscle weight was also maintained when mice were fed a ketogenic formula [31]. In the current work, female KPC mice that were fed a KD showed higher muscle weights compared CD fed ones. However, a KD did not further increase gastrocnemius muscle mass when gemcitabine was added. In fact, gemcitabine alone increased muscle mass without improving muscle strength, which could result in poorer clinical outcomes. We previously demonstrated a similar dissociation between changes in muscle mass and strength in old muscles following hind limb unloading [32]. In this instance, the lower strength in old muscles could be explained by impaired lateral force transfer, specifically by the loss of dystrophin [33]. In 2005, Archaryya et al. demonstrated that murine models show a similar decrease in dystrophin protein levels in C-26 tumor bearing mice, identifying a link between muscular dystrophies and CAC [34]. Since a KD was shown to improve muscle structure and function in a rat model of muscular dystrophy [35], a KD may improve muscle strength in part by increasing dystrophin levels and improving force transfer in this model of CAC.

Cachexia is, in part, driven by reduced appetite and a consequent decrease in caloric intake, and so, ameliorating cachexia-related anorexia is an important aspect of PDAC treatment [36]. We observed that anorexia, assessed by either food intake or caloric intake, was diminished in the KG groups when compared to CG. Anorexia was lessened in KG treated female mice, which could be a factor in the maintenance of muscle function. Interestingly, Koutnik et al. reported anti-anorexic effects of ketone diesters that are consistent with our data [37]. 

Although sex may play a role in cancer cachexia development and progression, preclinical studies elucidating sex differences in cancer cachexia are scarce [38]. Our data suggest that a KD results in preservation of skeletal muscle mass and function, and that response to a KD differs between females and males. Consistently, reduction in grip strength was shown to differ between female and male cachectic patients [39]. Both in animal models and human participants, CAC affects females and males differently. In a colorectal cancer model, female mice exhibited more severe reductions in body weight and muscle mass compared with male mice [40]. In general, females appear to be more susceptible to glucocorticoid-induced muscle atrophy [41], suggesting that corticosteroids may play a role in CAC and might be altered on a ketogenic diet.

Identifying drivers of cancer-associated muscle loss holds significant promise for improving cancer patient survival and quality of life. To begin addressing mechanism, the current work conducted a pilot analysis on the effect of diet on inflammation, mTORC1 signaling, and protein degradation. This pilot work showed that in females, a ketogenic diet tended to decrease inflammatory (NFκB phosphorylation) and AKT/mTORC1 signaling (AKT, s6, and 4E-BP phosphorylation) in the post-absorptive state. This finding is consistent with the idea that a ketogenic diet decreases NLRP3 inflammasome activity [42]. A decrease in mTORC1 activity may seem contrary to improved muscle mass and strength; however, it is important to note that muscles were collected in the post-absorptive state. If the activation of mTOR1 following feeding occurs to the same degree, this lower baseline would provide greater dynamic range and improved anabolic signaling. Another regulator of protein synthesis that was modulated by a KD was eIF2α. The phosphorylation of eIF2α increases with KG treatment (main effect of diet), with a stronger effect observed in females. The phosphorylated eIF2α decreases protein synthesis by inhibiting translation initiation. Phosphorylation of eIF2α is normally decreased in tumor-bearing mice [43,44], suggesting that eIF2α may have a paradoxical effect on muscle mass. Interestingly, we previously observed that p-eIF2α was higher in 26-month-old KD fed mice when compared to CD despite a significantly greater muscle mass [26]. Phosphorylated eIF2α in response to a KD may decrease translation initiation and improve proofreading, resulting in decreased unfolded proteins and less ER stress [26].

Autophagy is essential to maintaining skeletal muscle function [45]. Nevertheless, findings regarding markers of autophagy on a KD are equivocal, with reports showing that KD had no effect [26], or increased autophagy [46]. In our KPC mouse cohort, we observed a trend for higher LC3B-II to LC3B-I ratio in the muscle of KG-treated male mice, while the opposite effect occurred in KG-treated females. Since excessive autophagic degradation plays a role in the onset of muscle depletion in CAC [47], the inhibition of autophagy observed in our KPC female mice might be a factor in the sex-specific beneficial effects of KG treatment. 

Moreover, although the UPS is activated in SKM atrophy and muscle-wasting conditions, it also removes damaged proteins and helps maintain homeostasis [48]. We measured proteasome function in muscles from male mice from the CG and KG groups. Our data suggest that UPS activity shifts from caspase-like (β1) to trypsin-like (β2). Even though there was a diet-induced shift in proteosome activity, since more than half of total proteosome activity in muscle went through β5 (chymotrypsin-like activity), this was not changed by diet protein breakdown and was unlikely to drive the greater muscle mass on a KD. 

The regulation of gene expression in cachectic SKM can also be controlled by epigenetic mechanisms, through acetylation and deacetylation of histones, which are modified in a post-translational manner through histone acetyltransferases (HATs) and HDACs [49]. Since BHB can rapidly be converted into acetyl-CoA (the acetyl donor) and inhibit HDACs [27,28], we previously showed that a KD increases total acetylated lysine residues [26]. In the current study, we did not observe a difference in acetyl-lysine levels in the KG mice compared to CG. We previously showed that one, two or twelve months on a ketogenic diet increased total acetyl-Lys levels when compared to CD [12,26]. Why total acetyl-Lys levels and downstream effects like increases in PGC-1α and mitochondrial mass are not seen with concomitant treatment with a KD and gemcitabine is an interesting finding worthy of more research.

Some limitations of this study included the small sample size and the use of only GTN for the mechanistic study, and no other skeletal muscle tissues. Thus, additional research is warranted to further explore the mechanisms underlying the benefit of a KD plus gemcitabine on cachexia. Moreover, an area worth exploring is whether intermittent KD schedules can also provide a beneficial effect comparable to what was observed using a strict KD. This is significant, since adhering to a strict KD throughout PDAC treatment. might be difficult. Ultimately, future studies are needed to examine whether long-term co-adjuvant KDs can be clinically effective in combating CAC in individuals with PDAC. In summary, our findings indicate a beneficial effect of a KD in combination with gemcitabine in the preservation of skeletal muscle strength in pancreatic tumor bearing mice. The mechanisms underlying the favorable effect of a KD on CAC appears to be multifaceted, including decreased muscle inflammation, and increased eIF2α phosphorylation. 

## 4. Materials and Methods

### 4.1. Animal Studies

All animal use procedures were approved by the University of California, Davis Animal Care and Use Committee. All experiments were conducted in accordance with relevant guidelines and regulations, and they complied with the ARRIVE guidelines.

### 4.2. Genetically Engineered Transgenic Mice

The genetically engineered LSL-Kras^LSL-G12D/+^Trp53^R172H/+^Pdx-1-Cre (KPC) mice that model PDAC were bred at the UC Davis Animal Facility in Meyer Hall. The KPC mice were generated from three parental strains (LSL-Kras^G12D/+^, LSL-Trp53^R172H/+^, and Pdx-1-Cre) obtained from National Cancer Institute (NCI) mouse repository and following established procedures described by Hingorani and colleagues [50]. After weaning, rodents were individually housed in polycarbonate cages in a room with controlled temperature (22–24 °C) and humidity (40–60%), maintained on a 12 h light-dark cycle, and fed an ad libitum chow diet (LabDiet 5001, LabDiet, Saint Louis, MO, USA) until grouped for study. 

### 4.3. Survival Study

KPC mice were enrolled in the study after their tumor size was measured using a high-resolution ultrasound imaging of the pancreas using a Vevo 2100 System with a 35 MHz RMV scan-head (Visual Sonics, Inc., New York, NY, USA), following previously published guides [51,52]. After assessing tumor size, male and female KPC mice were allocated to either a control diet (CD: %kcal: 20% fat, 15% protein, 65% carb), a KD (%kcal: 84% fat, 15% protein, 1% carb), a CD + chemotherapeutic agent gemcitabine (CG), or a KD + gemcitabine (KG) group, as previously described [16]. All mice were fed ad libitum throughout the study, food was changed, and food intake was recorded three times per week. Gemcitabine (>99% 2′-Deoxy-2′,2′-difluorocytidine; dFdC; Gemzar; LY-188011) from Fisher Scientific (Hampton, NH, USA) was administered to the CG and KG groups at a dose of 100 mg/kg by intraperitoneal injection two times per week for 3.5 weeks (7 total injections). 

The composition of the experimental diets was previously reported [16]. The Envigo (Indianapolis, IN, USA) mineral mix TD94046 was used for the control diets and the TD79055 was used for the KD due to their lower carbohydrate contents. For both diets, TD40060 (vitamin mix) was used. 

Throughout the survival study, mice were observed daily for signs of weight loss; hemorrhagic ascites; or other indications of advanced disease including loss of thermoregulation, inactivity, and presence of malignant ascites. Endpoint criteria included the development of abdominal ascites, weight loss exceeding 20% of the initial weight, or extreme weakness or inactivity. When an animal reached the endpoint criteria, it was euthanized by carbon dioxide asphyxiation, blood was collected and tissues, including pancreatic tumors were dissected, weighed, and then stored in liquid nitrogen, RNA later, or 10% buffered formalin.

### 4.4. Mechanistic Study

The experimental design was previously described [16]. Briefly, male and female KPC mice were allocated to either the CG or the KG groups after tumor detection and euthanized two months after starting the diet. At the end of the 2 months, the gastrocnemius muscle (GTN) was dissected, weighed, and then stored in liquid nitrogen, RNA later, or 10% buffered formalin. The right GTN was collected to evaluate potential mechanisms involved in muscle strength preservation.

### 4.5. Forelimb Grip Strength Test

A forelimb grip strength dynamometer with electronic sensor (Columbus Instruments, Columbus, OH, USA) was used. Mice were allowed to grab the metal grid with forelimbs only and then pulled horizontally by the tail by the test administrator with even force until the animal let go of the bar. Two rounds of three trials each were performed with minimal rest between trials (1 min) and 30 min rest between rounds. The grip strength average of the six total trials was used for analysis. Force was measured in kg. Tests were performed at baseline and then monthly thereafter. 

### 4.6. Blood Glucose and Ketones

Non-fasting glucose levels were measured using a glucometer (Easy Plus II, Home Aid Diagnostics Inc., Deerfield Beach, FL, USA), and ketone levels were measured using the Precision Xtra glucose and ketone monitoring system (Abbot, Chicago, IL, USA) according to the manufacturer’s instructions. Blood was obtained by tail-tip cut.

### 4.7. Body Weight and Composition

Body weight was measured weekly. Body composition was assessed using NMR relaxometry (EchoMRI-100H, EchoMRI LLC, Houston, TX, USA) at baseline and then monthly thereafter.

### 4.8. Metabolic Measurements

Blood samples were collected via cardiac puncture and serum was isolated after centrifugation at 3000× *g* for 10 min at room temperature. Insulin and leptin were assayed using the V-PLEX mouse metabolic kit and mouse leptin kit. Inflammation-related biomarkers were assayed using the V-PLEX Proinflammatory panel I kit (Meso Scale Discovery, Rockville, MD, USA). 

### 4.9. Tissue Homogenization and Western Blotting

Frozen GTN was powdered on dry ice using a mortar and pestle. Two scoops of powder were then aliquoted into 1.5 mL Eppendorf tubes and homogenized in 200 μL of sucrose lysis buffer [1M Tris, pH 7.5, 1M sucrose, 1mM EDTA, 1mM EGTA, 1% Triton X-100, and 1X protease inhibitor complex]. The solution was set on a shaker for 60 min at 4 °C, centrifuged at 8000 times g for 10 min, supernatants were transferred to new Eppendorf tubes, and protein concentrations were determined using the DC protein assay (Bio-Rad, Hercules, CA, USA). Equal aliquots of 300 μg of protein were diluted in 4× Laemmli sample buffer (LSB) (final volume 150 µL) and boiled for 5 min at 100 °C. Ten μL of protein sample (20 µg) was loaded into each lane of a Criterion TGX Stain-Free Precast Gel and run for 45 min at a constant voltage of 200 V. To ensure the most accurate normalization, total protein for each individual lane was quantified using gel images using GeneSnap Software (version 7.12) after being exposed to UV light for one minute. This process ensured that the expression of all the proteins in each individual lane on the Criterion-TGX gels were quantified after the gel was run with the loaded samples and prior to transfer, and we used this process to normalize our immunoblot results [25]. Proteins were then transferred to an Immobilon-P PVDF membrane, after it was activated in methanol and normalized in transfer buffer, at a constant voltage of 100 V for 30 min. Membranes were blocked in 1% Fish Skin Gelatin (FSG) in TBST (Tris-buffered saline w/0.1% Tween) and incubated overnight at 4 °C with the appropriate primary antibody diluted in TBST at 1:1000. The next day, membranes were washed with TBST for 5 min, and subsequently incubated at room temperature for one hour with peroxidase-conjugated secondary antibodies (1:10,000) in a 0.5% Nonfat Milk TBST solution. Bound antibodies were detected using a chemiluminescence HRP substrate detection solution (Millipore, Watford, UK). Imaging and band quantification were determined using Bio-Rad Image Lab Software (Hercules, CA, USA).

### 4.10. 26S Proteasome Activity Assay

Briefly, pulverized GTN muscle samples (20 mg aliquot) were homogenized in 26S proteasome lysis buffer (50 mM Tris, 150 mM sodium chloride (NaCl), 1 mM EDTA, 5 mM magnesium chloride, 1 mM DTT (freshly added) [pH 7.5]) with a hand-held Potter-Elvehjem homogenizer on ice. The supernatant containing the proteasomes were separated after centrifugation at 12,000× *g* for 15 min at 4 °C and were quantified and diluted to 1 μg/μL concentration with proteasome lysis buffer. The β5 (chymotrypsin-like), β2 (trypsin-like), and β1 (caspase-like) activities were assayed using 20 µg of protein. The assays were carried out in a total volume of 100 µL per well in a black 96-well plate. The adenosine triphosphate (ATP)-dependent 26 S assays were performed using 100 μM ATP in the absence and presence of a specific proteasome inhibitor: 10 μM bortezomib (β5 activity) and 100 μM bortezomib (β1 and β2 activities). The assays were initiated by the addition of specific fluorescence-tagged substrates for each proteasomal β subunits (Enzo Life Sciences, New York, NY, USA): Z-LLE-AMC for β1, Boc-LSTR-AMC for β2, and Suc-LLVY-AMC for β5 [53,54,55,56]. The final concentration of substrate in each assay was 100 μM. Proteasomal activities were measured every 15 min up to 120 min at an excitation wavelength of 390 nm and an emission wavelength of 460 nm using an Infinite M1000 PRO fluorometer.

### 4.11. Statistical Analysis

The data, obtained from at least three independent samples, were expressed as the mean ± SEM. Statistical evaluation was performed by t-test, or one- or two-way analysis of variance (ANOVA) followed by the Tukey test adjusted for multiple comparisons. Analyses were performed by GraphPad (Prism version 9.2) and R version 4.0.4. Two-sided *p* < 0.05 was regarded as statistically significant.

For longitudinal outcomes measured repeatedly over time within the same animal, repeated measures ANOVA tests were performed. To adjust for potential confounders, linear mixed-effects regressions were also fitted for post-treatment repeatedly measured grip strength and ketone bodies, which included fixed effects of diet (ketogenic, control), drug (gemcitabine or not), time since treatment (in days, centered at 30 days), and their 2-way and 3-way interactions, baseline age (in days, centered at 90 days), sex, baseline weight, baseline tumor size per unit weight, baseline outcome, weight at the time of outcome measurement, and random intercepts to account for correlation within animal. Interactions were retained in the final models only if the tests for interactions (or the higher order interactions) were significant. Baseline outcome and baseline age were always adjusted in the models. Baseline tumor size per unit weight, sex (for ketone bodies), baseline and time-varying weights were removed due to no significance. Baseline outcomes were centered at 3.8 (g/g BW) for grip strength per unit weight, and 0.43 (mmol/L) for ketone level; thus, the intercept estimate can be interpreted as the predicted outcome at day 30 for a female animal taking control diet and no GEM, with baseline outcome at the centered value. The coefficient estimate for a continuous covariate (e.g., baseline age) can be interpreted as the average increase in outcomes for a unit increase in the covariate. 

## Figures and Tables

**Table 1:** Parameter estimates from the final linear mixed-effects models for outcome.

	Outcome			
Model Term	Grip Strength (per Unit of Weight) g/g BW		Ketone Bodies (mmol/L)	
	Estimate (SE)	*p*-Value	Estimate	*p*-Value
Intercept	3.04 (0.11)	<0.001	0.487 (0.067)	<0.001
Effect of KD vs. CD (without GEM, at day 30)	−0.17 (0.11)	0.121	0.128 (0.08)	0.115
Effect of GEM vs. No GEM (with CD, at day 30)	0.07 (0.093)	0.436	−0.148 (0.094)	0.121
Effect of Time since intervention (in days)(with CD and no GEM)	−0.02 (0.0039)	<0.001	−0.002 (0.003)	0.402
Effect of baseline age (in days)	−0.0042 (0.0036)	0.247	0.001 (0.002)	0.654
Effect of Male vs. Female	−0.35 (0.085)	<0.001	-	-
Effect of baseline outcome	0.14 (0.073)	0.066	0.016 (0.196)	0.935
Interaction between KD and GEM	-	-	0.355 (0.124)	0.006
Interaction between KD and Time	0.021 (0.0053)	<0.001	0.008 (0.004)	0.024
Interaction between GEM and Time	-	-	0.005 (0.004)	0.222
Interaction between KD, GEM, and Time	-	-	−0.018 (0.005)	0.002

Note: Linear mixed-effects regression models were fitted for repeatedly measured post-treatment outcomes, which included fixed effects for diet, drug, time, and their 2-way and 3-way interactions (if significant), baseline age, sex, baseline outcome, and random intercepts for animal. Abbreviations: CD: control diet; KD: ketogenic diet; GEM: Gemcitabine.

## Data Availability

The data used and analyzed in the current study are available from the corresponding author upon request.

## Supplementary Materials

The following supporting information can be downloaded at: https://www.mdpi.com/article/10.3390/ijms241310753/s1.
