# Supplementary material for: A Ketogenic Diet in Combination with Gemcitabine Mitigates Pancreatic Cancer-Associated Cachexia in Male and Female KPC Mice
Source: Int J Mol Sci. 2023 Jun 28;24(13):10753. doi: 10.3390/ijms241310753 (PMC10341838; doi:10.3390/ijms241310753)
Supplement: Supplementary file 1 [file ijms-24-10753-s001.zip › ijms-2418776-supplementary.pdf]

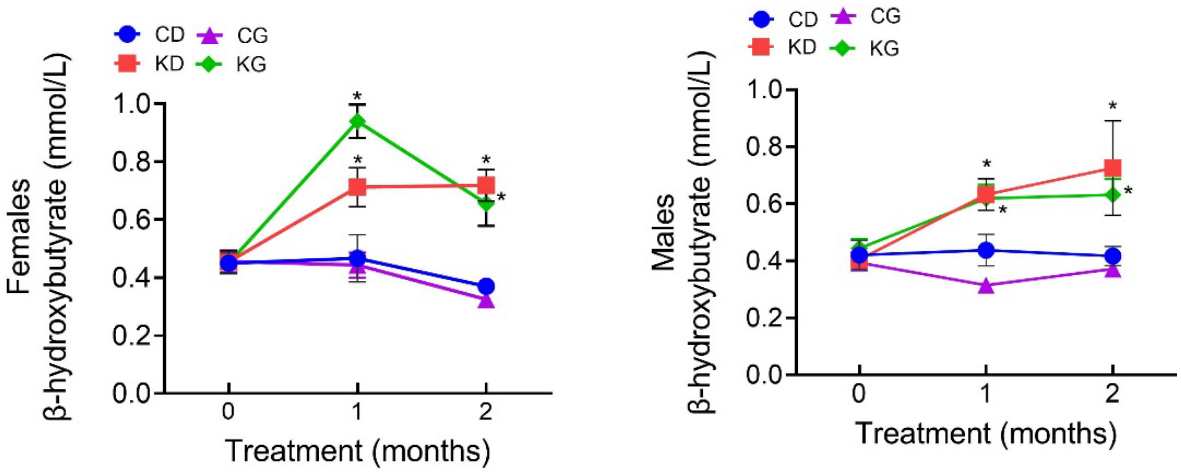

Supplemental Figure 1. Effect of a KD alone and in combination with gemcitabine on β-hydroxybutyrate levels in female and male KPC mice. Non-fasted blood β-hydroxybutyrate levels at baseline, one and two months after diet initiation are shown in KPC mice from the survival study cohort randomized to a control diet (CD), ketogenic diet (KD), CD plus gemcitabine (CG) or KD plus gemcitabine (KG); Values are expressed as means ± SEM; *p<0.05, ***p* < 0.01.

A

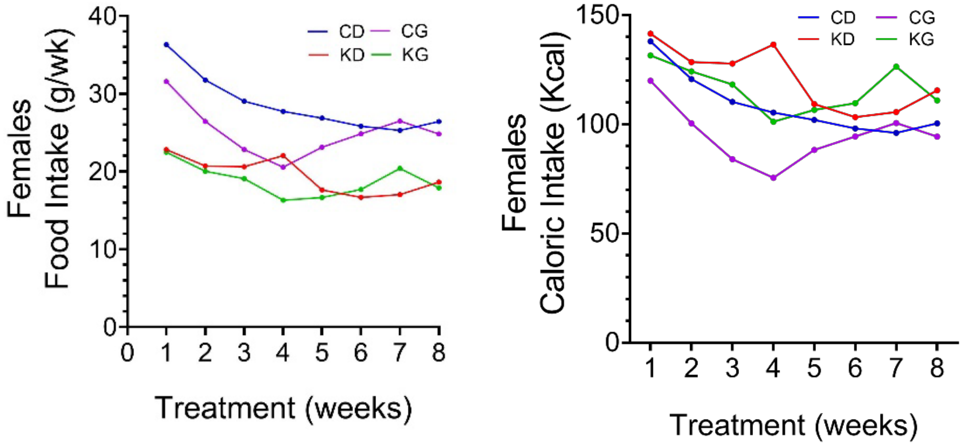

	FEMALES			
	CD	KD	CG	KG
Food intake (g/wk)	28.7 ± 1.3^{a}	$19.5 \pm 0.8^{a,b}$	$23.3 \pm 0.9^{a,b,c}$	$17.3 \pm 0.5^{a,c}$
Caloric intake (kcal/wk)	108.9 ± 5.0	121.0 ± 5.1^{b}	$94.7 \pm 4.7^{b,c}$	116.1 ± 3.8^{c}

B

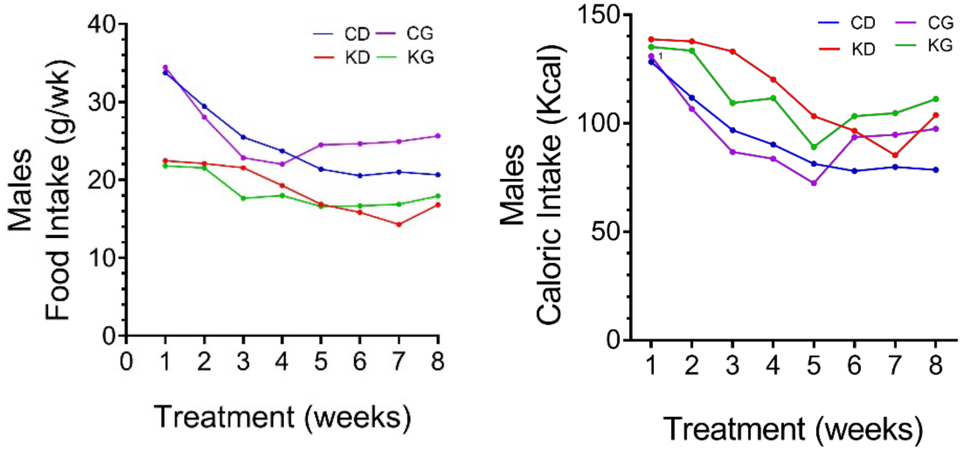

	MALES			
	CD	KD	CG	KG
Food intake (g/wk)	24.5 ± 1.7	18.6 ± 1.1	25.9 ± 1.4	18.4 ± 0.7
Caloric intake (kcal/wk)	93.1 ± 6.5	114.8 ± 7.2	95.7 ± 6.2	112.2 ± 5.4

Supplemental Figure 2. Effect of a KD alone and in combination with gemcitabine on food intake in female and male KPC mice. Food intake in grams per week (g/wk) and kilocalories per week (Kcal/wk) shown for **(A)** females and **(B)** males of the KPC mice from the survival study cohort randomized to a control diet (CD), ketogenic diet (KD), control diet plus gemcitabine (CG) or ketogenic diet plus gemcitabine (KG); Values are expressed as means ± SEM. Values having different superscripts are significantly different ($p < 0.05$).

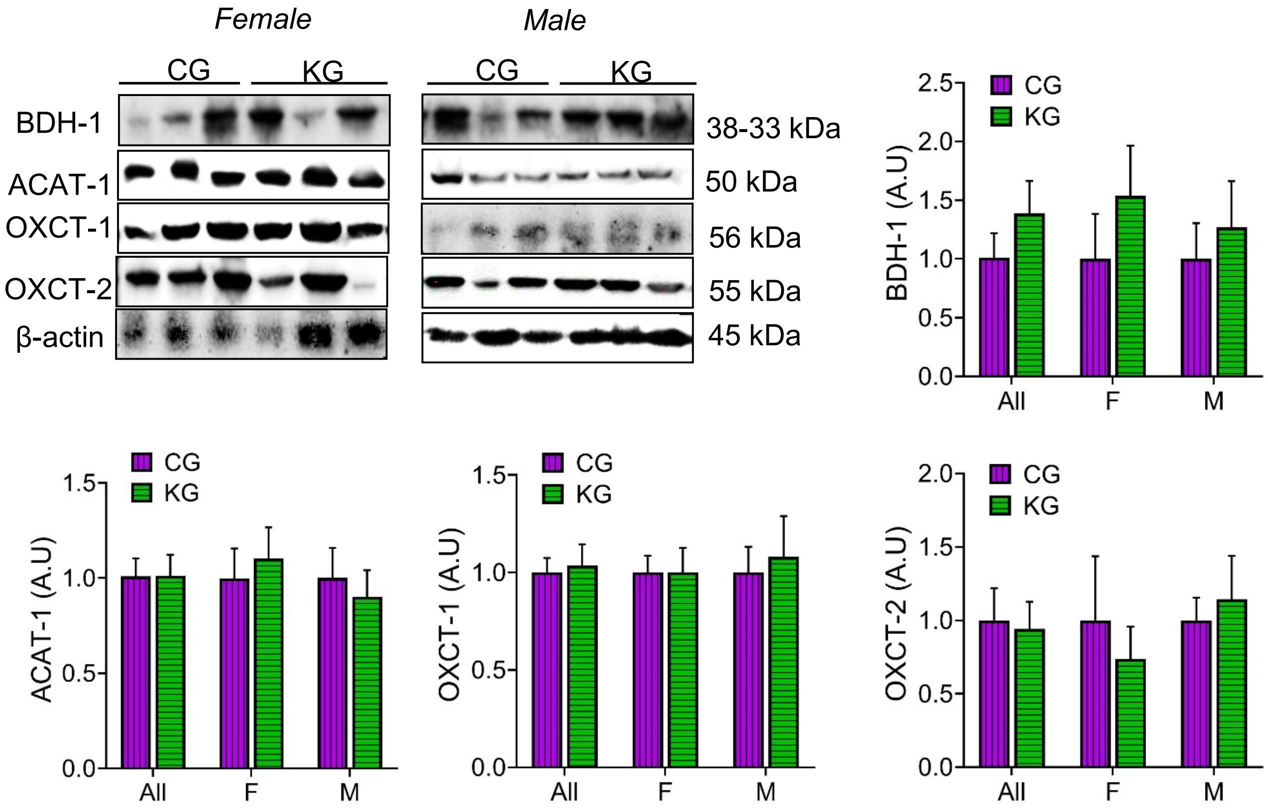

Supplemental Figure 3. Effect of a ketogenic diet in combination with gemcitabine on ketone body metabolic enzymes. Immunoblots of the ketone body metabolic enzymes succinyl CoA: 3-oxoacid CoA transferase (OXCT1), 3-hydroxybutyrate dehydrogenase 1 (BDH1), and acetyl-CoA acetyltransferase 1 (ACAT1) signaling from the gastrocnemius (GTN) of KPC mice treated with control diet plus gemcitabine (CG) or ketogenic diet plus gemcitabine (KG) separated by sex; *n*= 2-5; Values are expressed as means ± SEM.

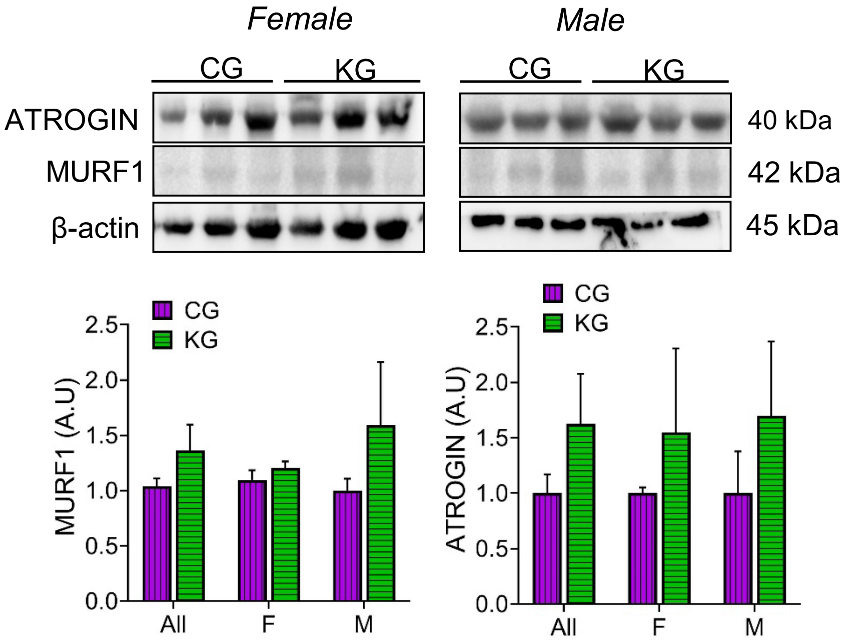

Supplemental Figure 4. Effect of a ketogenic diet in combination with gemcitabine on muscle-specific E3 ubiquitin ligases. Immunoblot of the muscle-specific E3 ubiquitin ligases atrophy-related gene (ATROGIN-1) and muscle RING finger 1 (MURF-1) signaling from the gastrocnemius (GTN) of KPC mice treated with control diet plus gemcitabine (CG) or ketogenic diet plus gemcitabine (KG) separated by sex; *n*= 2-5 per sex per group. Values are expressed as means ± SEM.
